# BabA dependent binding of *Helicobacter pylori* to human gastric mucins cause aggregation that inhibits proliferation and is regulated via ArsS

**DOI:** 10.1038/srep40656

**Published:** 2017-01-20

**Authors:** Emma C. Skoog, Médea Padra, Anna Åberg, Pär Gideonsson, Ikenna Obi, Macarena P. Quintana-Hayashi, Anna Arnqvist, Sara K. Lindén

**Affiliations:** 1 Department of Biomedical Chemistry and Cell Biology, Institute of Biomedicine, Sahlgrenska Academy, University of Gothenburg, Gothenburg, Sweden; 2 Department of Medical Biochemistry and Biophysics, Umeå University, Umeå, Sweden

## Abstract

Mucins in the gastric mucus layer carry a range of glycan structures, which vary between individuals, can have antimicrobial effect or act as ligands for *Helicobacter pylori*. Mucins from various individuals and disease states modulate *H. pylori* proliferation and adhesin gene expression differently. Here we investigate the relationship between adhesin mediated binding, aggregation, proliferation and adhesin gene expression using human gastric mucins and synthetic adhesin ligand conjugates. By combining measurements of optical density, bacterial metabolic activity and live/dead stains, we could distinguish bacterial aggregation from viability changes, enabling elucidation of mechanisms behind the anti-prolific effects that mucins can have. Binding of *H. pylori* to Le^b^-glycoconjugates inhibited the proliferation of the bacteria in a BabA dependent manner, similarly to the effect of mucins carrying Le^b^. Furthermore, deletion of *arsS* lead to a decrease in binding to Le^b^-glycoconjugates and Le^b^-decorated mucins, accompanied by decreased aggregation and absence of anti-prolific effect of mucins and Le^b^-glycoconjugates. Inhibition of proliferation caused by adhesin dependent binding to mucins, and the subsequent aggregation suggests a new role of mucins in the host defense against *H. pylori*. This aggregating trait of mucins may be useful to incorporate into the design of adhesin inhibitors and other disease intervention molecules.

The mucus layer that covers mucosal surfaces is the first barrier that gastrointestinal bacteria encounter. The mucus layer in the stomach consists mainly of MUC5AC secreted from the surface mucosa and MUC6 secreted from the gland mucosa^1^. In half of the human population, the surface mucosa of the stomach is colonized by the pathogen *Helicobacter pylori*, which can cause gastritis, gastric and duodenal ulcers, adenocarcinoma and MALT lymphoma^2^,^3^. *H. pylori* adheres to highly glycosylated mucins via the blood group antigen binding adhesin (BabA) that binds to Lewis b (Le^b^) and related fucosylated structures, and the sialic acid binding adhesin (SabA) that binds mainly to sialyl-Lewis x (SLe^x^) and sialyl-Lewis a (SLe^a^)^4^,^5^,^6^. Adhesion of *H. pylori* to mucins and host cells is highly relevant for colonization, mucin-mediated defense, host cell response and development of disease^7^,^8^,^9^,^10^. The adhesion targets and the glycan environment that *H. pylori* is exposed to vary between individuals and change as the expression, glycosylation and spatial distribution of mucins change in response to *H. pylori* infection and disease development^11^,^12^,^13^,^14^,^15^,^16^,^17^. For example, in the rhesus monkey (*Macaca mulatta*) infection model there are time-dependent changes in Le^b^ expression and in the same monkeys, as well as in mice, gerbils and humans, there is an induced expression of sialylated Lewis antigens upon infection with *H. pylori*^16^,^18^,^19^,^20^. However, the effect of a variably glycosylated environment on *H. pylori* is poorly understood.

We recently reported that the proliferation of *H. pylori* can be modulated differently by mucins from different individuals and disease states^21^. Mucins from some of the patients stimulated the proliferation of *H. pylori*, while mucins from other patients tended to have a growth repressing effect. The latter could not be explained solely by the presence of terminal α1,4-linked *N*-acetylglucosamine (α1,4-GlcNAc), which is a carbohydrate structure with antimicrobial activity present on mucins in the gastric glands^22^, as this structure was not detected in all mucin samples that repressed *H. pylori* proliferation. The binding ability of *H. pylori* to mucins is also of importance for proliferation, as *H. pylori* strains with different mucin binding abilities vary in their proliferative response to mucins^21^,^23^. Furthermore, interaction with mucins can modify the *H. pylori* expression of adhesin genes^21^. Taken together, this demonstrates that mucins can regulate the behavior of *H. pylori* beyond acting merely as a physical barrier and attachment site.

In the present study, we further investigated the relationship between binding and response to mucins. We used *H. pylori* strains with different binding abilities, including deletion mutants of the *babA* and *sabA* adhesin genes, and studied their binding in relation to proliferation and gene expression in response to mucins and glycoconjugates with the ligands Le^b^ and SLe^x^. In addition, we included an isogenic mutant lacking the histidine kinase sensor protein ArsS, as an *arsS* deletion mutant has been shown to have an increased expression of *sabA*^24^,^25^, and this mutant thus represents a model with potentially increased SabA dependent binding. Combining measurements of optical density, bacterial metabolic activity and live/dead stains enabled elucidation of mechanisms behind the anti-prolific effects of mucins, and we can conclude that *H. pylori* ligand binding via BabA causes a decrease in proliferation, which appears related to the extent of aggregation caused by binding.

## Results

### Accurate interpretation of *H. pylori* proliferation and viability requires careful method selection

**Figure 1:**
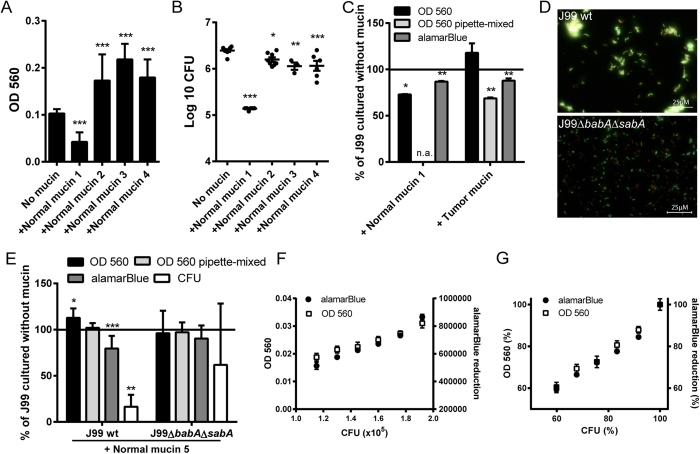
Proliferation and viability in response to different mucins. (**A**) OD_560_ of liquid cultures of *H. pylori* J99 cultured for 60 h with purified human gastric mucins from several individuals. *H. pylori* were still in the growth phase at this time point (i.e. had not entered the stationary phase). (**B**) CFU count of *H. pylori* J99 cultured for 60 h with human mucins. (**C**) Comparison between metabolic activity, measured as reduced alamarBlue, and OD_560_ of *H. pylori* J99 cultured for 24 h with or without the normal mucin sample 1 (does not induce formation of aggregates, but carries the α1,4-GlcNAc structure that has antibiotic like properties) and the tumor mucin sample (induce formation of aggregates and does not carry α1,4-GlcNAc). Cultures were shaken or pipette-mixed prior to OD_560_ measurement. Values are shown as percentage of J99 cultured without mucins (represents 100%), n.a. = not analyzed. (**D**) LIVE/DEAD *Bac*Light staining of the J99 wt and J99Δ*babA*Δ*sabA* deletion mutant after culture with Le^b^-positive mucins isolated from a normal stomach (normal mucin 3). Live bacteria are colored green and dead bacteria are colored red. (**E**) Comparison between OD_560_ measurements, metabolic activity and CFU counts for J99 wt and J99Δ*babA*Δ*sabA* deletion mutant after culture with Le^b^-positive mucin sample 5. Values are shown as percentage of each strain cultured without mucins (represents 100%). (**F**) The relationship between the alamarBlue signal, CFU counts and OD_560_ in the absence of mucin. (**G**) The relationship between the % change in alamarBlue signal at OD_560_, CFU counts and OD_560_ in the absence of mucin (100% corresponds to the highest value in (**F**). All values are presented as mean ± S.E.M, *p ≤ 0.05, **p ≤ 0.01, ***p ≤ 0.001: statistical tests were performed with ANOVA with Dunnett’s post hoc test, except the CFU data in panel E, which was analyzed with the Mann-Whitney U test.

Optical density is a widely used measure of bacterial growth, and has been used both by us and other research groups previously with the interpretation that the OD value of bacteria cultured with mucins is directly related to the cell count^21^,^22^. Although we have verified by CFU counts that for a subset of mucin-bacterial samples this appears accurate, we have now found that several cultures of bacteria with strong binding to the added mucins resulted in high OD_560_ values (reading performed after vigorous manual shaking), despite low CFU counts (Fig. 1A, B and E). Therefore, we investigated the relationship of these parameters to develop a method for accurate assessment of proliferation and viability. We found that adhesin dependent bacterial binding to mucins isolated from a gastric tumor (positive for both Le^b^ and SLe^x^) and from surface mucosa of normal gastric tissue (normal mucin 3, positive for Le^b^) results in the formation of aggregates (Skoog *et al*.^21^ and Fig. 1D) along with high OD_560_ readings (Fig. 1A and C). Dispersion of the aggregates by thorough pipette mixing reversed the increased OD in the *H. pylori* culture with the tumor mucin, thereby demonstrating that the aggregates were the cause of the increased OD (Fig. 1C). In contrast, when *H. pylori* J99 were cultured with a mucin rich in the anti-microbial glycan structure α1,4-GlcNAc, the OD_560_ correlated with the CFU count and indicated fewer bacteria than without mucins, even though cultures were only mixed by shaking before OD_560_ measurement (Fig. 1C). Microscopy analysis of bacteria cultured with mucins indicated that aggregation is caused by specific adhesin-ligand interaction, as most *H. pylori* J99 wt were present in large clusters whereas the vast majority of J99Δ*babA*Δ*sabA* were unassociated (Fig. 1D). Aggregates may cause inaccurate results if not fully dispersed prior to the plating for CFU, as several bacteria in one spot would appear as one colony. Thus, as a measure of bacterial count, aggregation may cause an enhancing error in the OD_560_ measurements, but a diminishing error by the CFU counting method. The metabolic activity (which can be used as a measure of cellular viability) can be measured by adding alamarBlue to the cultures^26^. Both the metabolic activity (alamarBlue signal) and the OD_560_ readings after vigorous pipetting were lower after culture with the tumor mucin compared to culture without mucins (p < 0.05, Fig. 1C), and the alamarBlue thus seems to accurately measure *H. pylori* proliferation even though cultures were only mixed by shaking before and after the addition of alamarBlue. Using a mucin that was positive for Le^b^ and devoid of α1,4-GlcNAc (normal mucin 5), the OD_560_ increased, but was reversed after vigorous pipetting, and both the alamarBlue signal and the CFU counts decreased in cultures with J99 wt, whereas none of these parameters were affected in cultures with J99Δ*babA*Δ*sabA* (Fig. 1E). The relationship between the alamarBlue signal and CFU counts is similar to the relationship between OD_560_ and CFU counts in the absence of mucins (Fig. 1F): within the concentrations the experiments were performed, a % change in alamarBlue approximately equals the same % change in CFU (Y = 0.9399*X + 1.774, Fig. 1G). In summary, both OD and CFU counts, as a measure of bacterial proliferation, can be misleading when molecules that aggregate bacteria are present. Extrapolating metabolic activity into viability and proliferation appears more accurate in this context although one cannot separate proliferation and viability from each other. Microscopic examination of stains determining the number of live vs. dead cells can add additional information, but are time consuming and impractical if large sample numbers are to be analyzed simultaneously. Therefore, we mainly used alamarBlue to assay proliferation of *H. pylori* throughout the rest of this study, with microscopic examination on a subset of samples to visualize aggregation.

### Binding of *H. pylori* to Le^b^-glycoconjugates inhibits the proliferation of the bacteria, similarly to the effect of mucins carrying Le^b^

**Figure 2:**
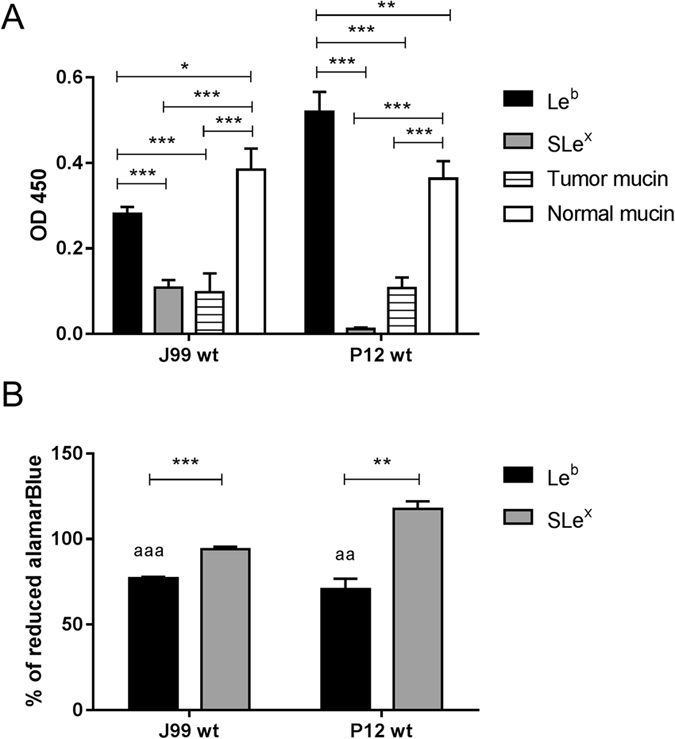
Proliferation and adhesion of J99 and P12 in response to Le^b^- and SLe^x^-glycoconjugates. (**A**) Binding of J99 and P12 to Le^b^- and SLe^x^-glycoconjugates and to the tumor and normal mucin sample analyzed using the microtiter plate based assay (n = 4). (**B**) Analysis of the metabolic activity as the percentage of reduced alamarBlue in relation to parallel cultures without glycoconjugates (represents 100%) demonstrated a loss of viability in the cultures with Le^b^-glycoconjugates (n = 6). Values are mean ± S.E.M, *p ≤ 0.05, **p ≤ 0.01, ***p ≤ 0.001. ANOVA with Bonferroni’s post hoc test or Student t-test, ^aa^p ≤ 0.01, ^aaa^p ≤ 0.001 ANOVA with Bonferroni’s post hoc test, compared to proliferation in the absence of glycoconjugates. The results in the graphs have been reproduced at least twice with the same outcome.

*H. pylori* binding to mucins that carry Le^b^ and SLe^x^, can inhibit proliferation (Fig. 1B,C and E)^21^. However, mucins carry a large repertoire of glycans and may also carry structures that can enhance growth^21^. Furthermore, strains may potentially vary in how they respond to glycan elements due to differences in genetic composition and thereby glycan response machinery. To study proliferation of *H. pylori* in response to individual glycans, we focused on strains J99 and P12. Both strains carry the *babA* gene, and J99 also carry the *sabA* gene. Strain P12 carries two *sabB* alleles^27^, which is a result from gene conversion with *sabA*^28^. SabB has an unknown function and thus P12 lacks the SabA mediated binding. Binding of both J99 and P12 was more pronounced to Le^b^ than to SLe^x^ (p < 0.001, Fig. 2). Furthermore, both J99 and P12 bound better to the mucin derived from a healthy stomach that carried Le^b^ (but not SLe^x^) than to the tumor derived mucin that carried both Le^b^ and SLe^x^ (p < 0.001, Fig. 2A). To study proliferation in response to individual glycans, we cultured strains J99 and P12 with Le^b^ and SLe^x^ conjugated to human serum albumin. Since adhesion to the SLe^x^-conjugate was either low or absent, the SLe^x^-glycoconjugate represents a glycan attached to the same carrier as the Le^b^-glycoconjugate but that does not cause major aggregation. Measurements of the metabolic activity of *H. pylori* by alamarBlue reduction demonstrated that Le^b^-conjugates decreased the metabolic activity of J99 and P12 (p < 0.05, Fig. 2B). Thus, binding of *H. pylori* to Le^b^-glycoconjugates inhibits the proliferation of the bacteria, similarly to the effect of mucins carrying these glycans. In contrast, the metabolic activity of P12 was enhanced in the presence of SLe^x^, which might reflect growth stimulation in response to glycans via an unknown mechanism that is independent of SabA and adhesion, as a similar growth enhancement was obtained in response to mucins in the absence of binding with this strain (below).

### The proliferation of the *babA* deletion mutants is not inhibited by Le^b^-positive mucins and glycoconjugates

**Figure 3:**
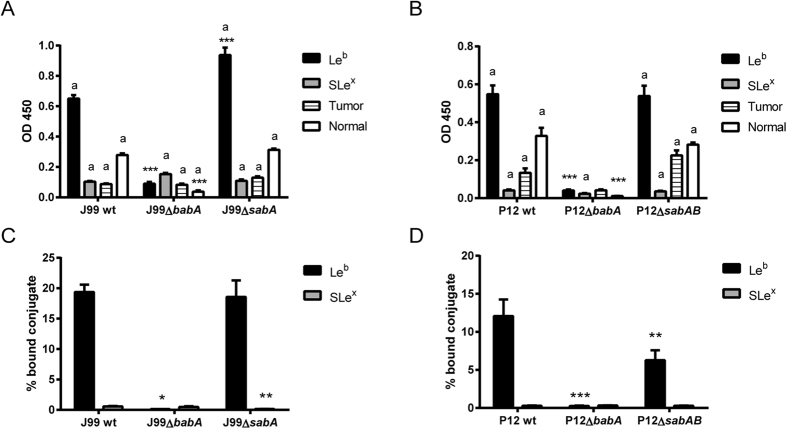
Binding of J99 and P12 wt and isogenic adhesin mutants to glycoconjugates and mucins. Binding of strain J99 (**A**) and P12 (**B**) to Le^b^- and SLe^x^-glycoconjugates, the tumor derived and normal mucin coated onto microtiter plates (n = 4). The results in the graphs have been reproduced at least twice with the same outcome. Values are mean ± S.E.M. Stars indicate statistical relationship of mutant binding compared to the binding of the isogenic wt to the same ligand, One-way ANOVA with Bonferroni’s post hoc test, *p ≤ 0.05, **p ≤ 0.01, ***p ≤ 0.001, whereas the letter a indicates that the binding (depicted after subtraction of the background signal) is statistically different (p < 0.05) from the background signal. Binding of strain J99 (**C**) and P12 (**D**) to Le^b^- and SLe^x^-glycoconjugates in solution using RadioImmuno assay (n = 9–10). Values are median ± interquartile range. Stars indicate statistical relationship of mutant binding compared to the binding of the isogenic wt to the same ligand, Kruskal-Walllis One-way ANOVA on ranks with Dunn’s multiple comparisons test, *p ≤ 0.05, **p ≤ 0.01, ***p ≤ 0.001.

To verify that *H. pylori* proliferation is inhibited by the glycan binding event specifically, we used adhesin mutants. These were cultured with glycoconjugates as well as with mucins to provide a more complex, *in vivo* like, glycan environment. In line with the genetic modification, the J99 and P12 *babA* deletion mutants (Δ*babA*) bound less to Le^b^-glycoconjugates and less to mucins isolated from a healthy stomach than the isogenic wt strains (Fig. 3). P12 binding to SLe^x^ was very low as expected due to the lack of *sabA*, and no difference in binding to SLe^x^ was detected with a P12 mutant where the *sabB* allele in the *sabA* locus had been deleted (Fig. 3B and D). Binding of J99 wt to SLe^x^ was also very low, but although no decrease in binding to SLe^x^ was detected with the J99 *sabA* deletion mutant (Δ*sabA*) using the microtiter based assay, a decrease was detected when binding was measured in solution in the presence of isotopes (RIA) (Fig. 3A and C). Thus, both strains mainly interact with mucins and glycoconjugates via BabA, but we still kept the J99Δ*sabA* in the below assays as an additional control to verify that the differences between wt and Δ*babA* strains were not due to procedures associated with the genetic manipulations or clone selection.

**Figure 4:**
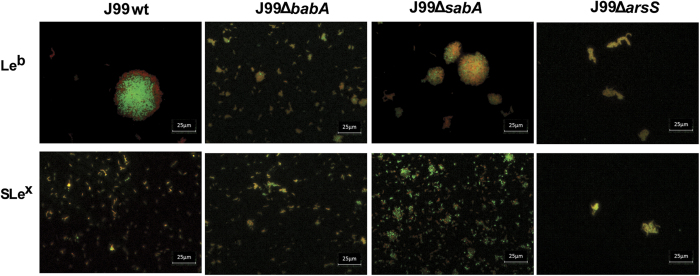
Images of aggregates formed after culturing J99 wt and its isogenic *arsS, babA* and *sabA* deletion mutants with glycoconjugates. Live (green color) and dead (red color) J99 wt, J99Δ*arsS,* J99Δ*babA and* J99Δ*sabA* after 24 h culture with Le^b^- and SLe^x^-glycoconjugates and stained with the LIVE/DEAD *Bac*Light bacterial viability kit. The images are representative of the whole sample, and of three experiments.

**Figure 5:**
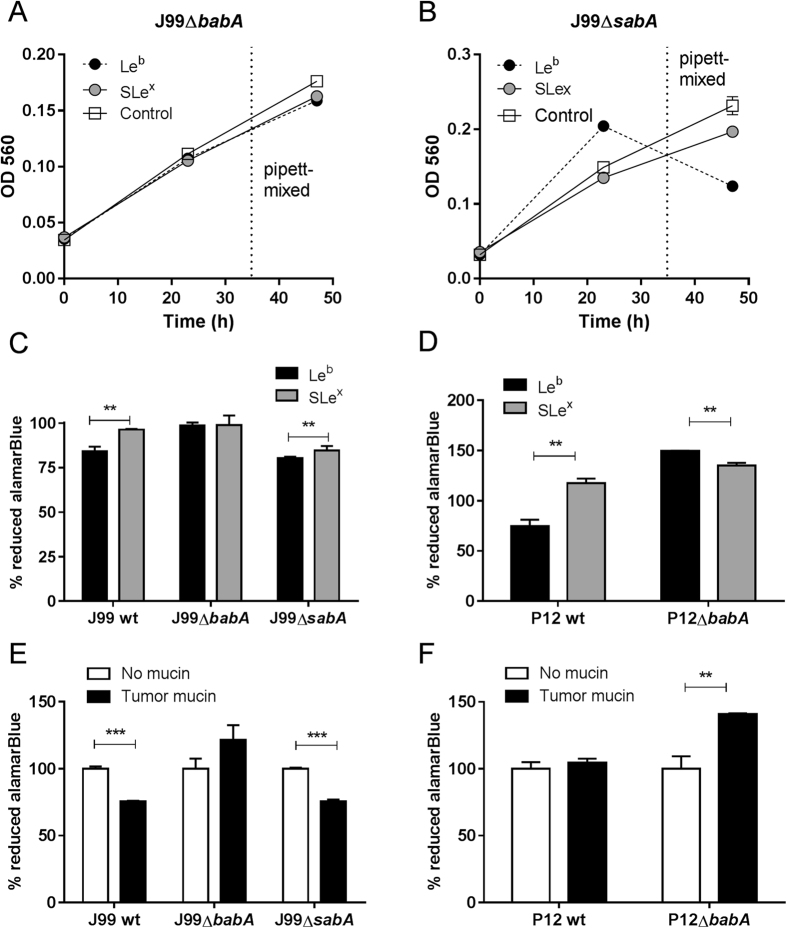
Proliferation of J99 and P12 and isogenic adhesin mutants after culture with glycoconjugates and mucins. (**A,B**) OD_560_ of J99Δ*babA* and J99Δ*sabA* during culture in the presence Le^b^- and SLe^x^-glycoconjugates. Endpoints of cultures were mixed by pipetting to break aggregates. (**C,D**) Viability of *H. pylori* after 24 h culture in the presence of the glycoconjugates as measured by alamarBlue reduction. (**E,F**) Viability of bacteria after 24 h culture in the presence of the tumor mucin sample as measured by alamarBlue reduction. All values are mean ± S.E.M, **p ≤ 0.01, ***p ≤ 0.001, Student’s t-test, n = 6. The results in the graphs have been reproduced at least twice with the same outcome.

J99Δ*sabA* as well as J99 wt formed aggregates in the presence of both the Le^b^-glycoconjugate (Fig. 4) and the tumor mucin sample (tumor mucin referred to as P1 TS in Skoog *et al*.^21^) resulting in an increase in OD (Figs 1C and 5B). When the bacteria were mixed by pipetting to break aggregates, the ODs at the end point of these cultures were instead decreased (Figs 1C and 5B), which corresponded to a decreased metabolic activity as measured by alamarBlue reduction (p < 0.001, Fig. 5C and E). The loss of proliferation in response to the Le^b^-glycoconjugate and the tumor mucin sample was reversed by deletion of *babA*, which also resulted in a decrease in formation of aggregates (Figs 4 and 5A,C,E: an image of the aggregates formed after culture of J99 with the tumor mucin has been published ref. 21). These results are in line with the OD and metabolic activity of J99 wt and J99Δ*babA*Δ*sabA* after culture with the normal mucin 5 in Fig. 1E. Similarly, the decreased metabolic activity of strain P12 wt in the presence of Le^b^, was reversed by deletion of *babA* (Fig. 5D). The tumor mucin did not have negative effects on the growth on strain P12, however, deletion of *babA* lead to enhanced growth in the presence of the mucin and Le^b^ (Fig. 5F), further indicating that this strain has a positive growth response to mucin glycans, which is suppressed by the BabA dependent binding to the mucin. Thus, this mucin has no negative effect on proliferation other than that caused by BabA dependent binding to Le^b^ (and similar structures) present on the mucin.

### Binding to Le^b^ affect adhesin gene expression

**Figure 6:**
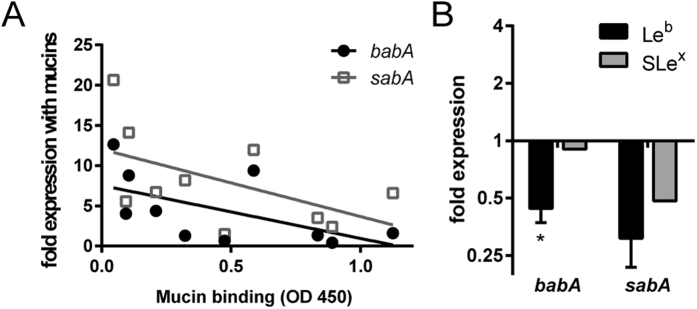
Expression of *babA* and *sabA* in response to mucins and ligand binding. (**A**) Correlation between mucin binding of J99 wt (as analyzed by microtiter based assay) to 10 different (individual) human mucin samples and gene expression of *babA* (p = 0.068, r = −0.597) and *sabA* (p = 0.085, r = −0.571, Pearson correlation) in J99 wt after 24 h culture with these mucins. 9 of the 10 mucins were positive for Le^b^ 21. (**B**) Expression of *babA* and *sabA* in *H. pylori* J99 after 24 h culture with Le^b^- and SLe^x^-glycoconjugates. Stars indicate change of expression compared to untreated bacteria confirmed by paired sample t-test, where Ct values of each experiment were paired to remove confounding factors of variable base levels between experiments. Fold expression compared to bacteria cultured without glycoconjugates or mucins are shown where expression is calculated as ΔCt in relation to expression of 16S rRNA as a housekeeping gene. Values are mean ± S.E.M. from 2 biological replicates, where each data point consists of the mean of 2 technical replicates with very similar result, *p ≤ 0.05.

We have previously shown that *H. pylori* adhesin expression can be differentially affected in culture with different mucins^21^. Here we investigated the relationship between binding to mucins and expression of the BabA and SabA adhesins in *H. pylori* J99 after co-culture with mucins, including mucins isolated from 10 individuals, and found that the Pearson product-moment correlation coefficient tended to be negative (p = 0.068, r = −0.597 and p = 0.085, r = −0.571, Fig. 6A). All samples contained MUC5AC and some also MUC6, and all but one sample were positive for Le^b^, while only two samples were positive for SLe^x^ 21. The very complex environment provided by the mucins carrying in the order of 100 different carbohydrate structures has potential to affect binding and stimulate the bacteria in multiple ways. Thus, even in this very complex surrounding, the overall effect of binding appears to be downregulating the adhesins. Therefore, we examined if the expression of the adhesins was affected by binding to its ligand after 24 h of culture with the SLe^x^- and Le^b^-glycoconjugates in strain J99. *babA* mRNA levels decreased after culture in the presence of Le^b^ (p < 0.05), but not in the presence of SLe^x^ (Fig. 6B), and expression of *sabA* trended towards being decreased after culture with Le^b^ and SLe^x^ (p = 0.09, Fig. 6B).

### Absence of ArsS reduces BabA dependent decreases in viability and aggregation

**Figure 7:**
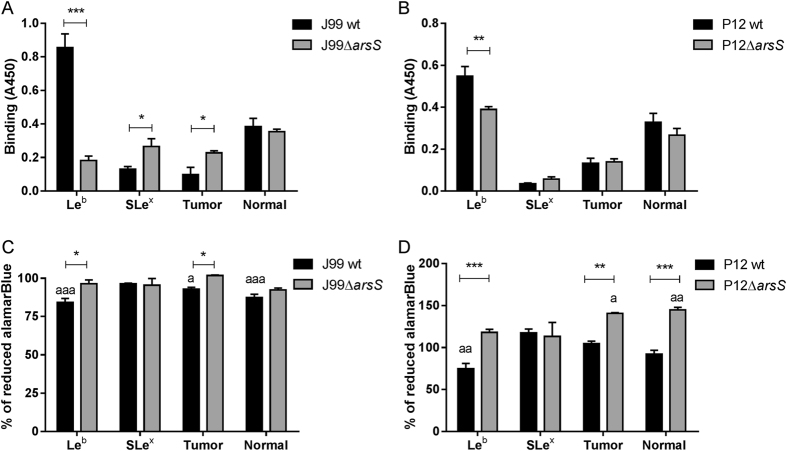
Binding of J99Δ*arsS* and P12Δ*arsS* to mucins and glycoconjugates and their viability in response to these mucins and glycoconjugates. (**A,B**) Binding of bacteria to Le^b^, SLe^x^, tumor mucin and normal mucin, using the microtiter based assay (n = 4). (**C,D**) Viability of bacteria after 24 h culture in the presence of Le^b^, SLe^x^, tumor mucin or normal mucin samples expressed as % change in alamarBlue reduction (n = 6). Results are shown as percent of reduced alamarBlue compared to each bacteria cultured without glycoconjugates/mucins, ^a^p ≤ 0.05, ^aa^p ≤ 0.01, ^aaa^p ≤ 0.001 ANOVA with Dunnett’s post hoc test, *p ≤ 0.05 **p ≤ 0.01, ***p ≤ 0.001 Student’s t-test. All values are mean ± S.E.M. The results in the graphs have been reproduced at least twice with the same outcome.

**Figure 8:**
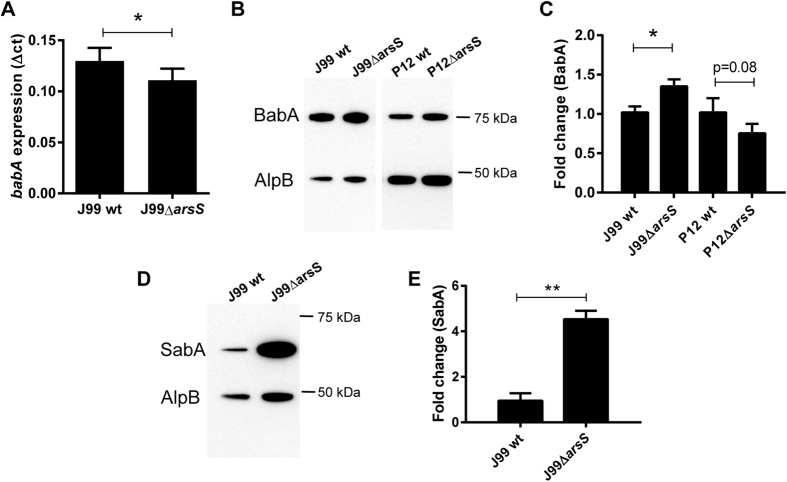
Effect of *arsS* deletion on BabA/*babA* expression. (**A**) mRNA expression of *babA* in J99 wt and J99*ΔarsS*. Expression of *babA* after 24 h of culture in liquid medium, measured as ΔCt in relation to 16 S rRNA as a housekeeping gene. Stars indicate difference in expression compared by paired sample t-test, *p ≤ 0.05, where Ct values of each experiment were paired to remove confounding factors of variable base levels between experiments (n = 3). (**B**) Immunoblot detecting BabA in bacterial lysates after growth on plate. AlpB was used as a loading control. (**C**) Quantification of the BabA immunoblot, normalized to AlpB (n = 5–7). (**D**) Immunoblot detecting SabA in J99 lysates. AlpB was used as a loading control. (**E**) Quantification of the SabA immunoblot, normalized to AlpB (n = 5–8). Data are presented as median with interquartile range, and analyzed with the Mann-Whitney U-test (*p ≤ 0.05, **p ≤ 0.01).

The level of SabA expression has previously been shown to be increased in a *H. pylori* strain with deleted *arsS* compared to its isogenic wt strain^24^. Consistent with these results, J99Δ*arsS* binding to SLe^x^ slightly increased compared to that of J99 wt (p < 0.05, Fig. 7A). Unexpectedly, binding to Le^b^ was decreased by 80% in J99Δ*arsS* (p < 0.0001, Fig. 7A), and by 30% in strain P12Δ*arsS* (p < 0.01, Fig. 7B), as determined using the microtiter based assays. In line with these results, binding to the mucin derived from the gastric tumor (carrying both Le^b^ and SLe^x^) increased in J99Δ*arsS* compared to J99 wt strain (p < 0.05), and binding to mucins derived from a healthy stomach (carrying Le^b^) trended towards a decrease in the P12Δ*arsS* strain. That the differences are less distinct with the mucins may be explained by the presence of a multitude of other structures that might contribute to binding. The decrease in binding to Le^b^ was accompanied by a slight decrease in *babA* gene expression in J99Δ*arsS* compared to wt (p < 0.05, Fig. 8A), but BabA protein expression was slightly increased in J99Δ*arsS* and tended to be decreased by a similar degree in P12Δ*arsS* (Fig. 8B and C, p < 0.05 and p < 0.08). In comparison, the level of SabA protein increased 6-fold in J99 Δ*arsS* compared to wt (Fig. 8D and E, p < 0.01). Together with that we found a decreased binding to Le^b^ conjugates in solution with the P12Δ*arsS* compared to wt (i.e. consistent with the microtiter based assay) but not for J99Δ*arsS* (data not shown) and that ArsS has been shown to be involved in protein trafficking^29^, the results indicate that it is the topographical localization or presentation of the adhesin that determines the level of BabA-dependent binding on microtiter plates and aggregation in solution (Fig. 5), and not the amount of adhesin present.

When cultured with Le^b^ or the tumor or normal mucin sample, the metabolic activity of J99 wt decreased (p < 0.05) whereas that of J99Δ*arsS* was not affected (Fig. 7C). This could be explained by the lesser formation of aggregates (Fig. 4), causing less hindrance to proliferation. In contrast, neither J99 wt nor J99Δ*arsS* proliferation was affected by SLe^x^. Similarly, the metabolic activity of P12 wt, but not of P12Δ*arsS,* decreased when cultured with Le^b^, and proliferation of P12Δ*arsS* was higher than that of the wt when cultured with mucins too (Fig. 7D). In line with that glycans can act stimulatory on this strain in the absence of *babA* dependent inhibition (Fig. 5F), the mucins enhanced growth of P12Δ*arsS.* The amplitude of the effects of deleting *arsS* on binding and proliferation differed between the strains, but decreasing BabA mediated aggregation via *arsS* deletion provides a second line of evidence for that adhesion dependent aggregation leads to decreased proliferation.

## Discussion

Here, we demonstrated that BabA dependent binding of *H. pylori* to Le^b^-glycoconjugates inhibits proliferation of the bacteria due to formation of aggregates, similarly to the effect of human gastric mucins carrying Le^b^, and that these events are regulated by ArsS. Furthermore, binding to Le^b^ decreases *babA* gene expression, indicating a negative feedback loop of the process. Thus, the results show that mucin-pathogen binding can have effects beyond merely inhibiting bacterial adhesion to the epithelial cell surface. The inhibition of proliferation caused by mucin binding and the subsequent aggregation suggests a new role of mucins in the host defense against *H. pylori*.

*H. pylori* have been observed by others to grow in aggregates within the human gastric mucus layer^30^. Growth limiting aggregation caused by mucin binding provides an additional manner for the host to control pathogen numbers alongside with that binding facilitate washing away the pathogen with mucus shedding. Formation of *H. pylori* aggregates in liquid cultures with mucins has also been reported by others^31^, who discuss that bacteria in aggregates may be protected from the outside environment. Similarly, *Pseudomonas aeruginosa*, has been shown to grow in aggregates within mucin gels, where aggregated bacteria are more resistant to antibiotics than non-aggregated bacteria of the same strain^32^. Thus, aggregation may also be beneficial for the bacteria protecting it from antibiotics, gastric acid and other harmful factors in their surroundings. Antibiotic resistance of *H. pylori* is a pressing issue^33^, and bacteria in aggregates may be exposed to subtherapeutic concentration of antibiotics, preventing fast eradication and enabling time to develop resistance. Disrupting aggregates formed in the gastric mucosa during eradication therapy might lead to a higher sensitivity to antibiotics and slower development of resistance.

As the majority of the bacteria were alive after culture with aggregate-causing mucins and glycoconjugates, as demonstrated by live/dead staining, there appears to be little or no direct antimicrobial activity caused by binding. The explanation for this might be that the binding causes formation of aggregates that slow down the proliferation due to physical hindrance or inter-bacterial communication. However, mucins are highly complex molecules, carrying in the order of 100 different glycan structures with potential to cause a multitude of effects. e.g. the ratio of live/dead bacteria was reduced in cultures with some mucins samples independent of aggregation, accompanied by a lower CFU count and reduced OD. These mucin samples are thus able to inhibit the proliferation via antimicrobial mechanisms, in analogy with the previously described “natural antibiotic” effect of terminal α1,4-linked *N*-acetylglucosamine^22^, which is present on some mucins. In addition, some mucin samples used in our previous studies increased the OD in the absence of noteworthy aggregation formation^21^. These samples are likely able to stimulate proliferation as suggested, similarly to the effect on strain P12, that we here show grows faster in the presence of glycoconjugates and mucins, when the growth inhibiting BabA dependent binding to Le^b^ is absent. The enhancement in growth could possibly be explained by the use of mucin glycans as a nutrient source or by activation of growth stimulating signaling pathways, although no such abilities have yet been described for *H. pylori. H. pylori* can thus be differently affected by mucins with different glycosylation. The majority of the patient mucins that we have investigated cause effects on growth ranging from −40 to +40%. Although these may appear to be marginal changes compared to for example antibiotics, *H. pylori* is a slow growing bacteria, present in relatively low numbers in most stomachs. Since infection often is present for 15–20 years before symptoms arise, these effects are likely to have major impact over time, in analogy to compound interest. Stimulation and inhibition of *H. pylori* proliferation by mucins from different individuals might be contributing factors to the outcome of the infection.

Another novel finding was that deleting *arsS* lead to a decreased aggregate formation. Growth of the *arsS* mutants were not inhibited in the presence of Le^b^-positive mucin and glycoconjugate compared to the corresponding isogenic wt strains, further supporting that it is the aggregation *per se* that causes the inhibition. There was only a 15% decrease in *babA* expression in J99Δ*arsS* compared to wt (p < 0.05), which does not seem proportional to the loss in aggregation, and similarly there were only marginal effects on BabA protein levels. Alternative mechanism for the decrease in binding and aggregation remains to be investigated, but our opinion is that altered surface presentation or accessibility due to changes in outer membrane protein composition (such as the increased expression of SabA) are the likely causes for the decreased aggregation. *arsS* deletion may also interfere with downstream signaling in response to aggregation.

As mucins can affect the proliferation of *H. pylori*, a rational hypothesis is that mucins also can affect other aspects of the bacteria, such as the expression of genes relevant for colonization and virulence. Indeed, it has previously been shown that the attachment to mucins and host cells can regulate *H. pylori* virulence genes^21^,^34^,^35^. Furthermore, the expression of two virulence factors (*cagA* and *ureA*), that were increased in the J99 wt strain after culture with mucins, was not affected in the isogenic mutant lacking the *babA* and *sabA* adhesins^21^. In this study we have investigated how the binding to the Le^b^- and SLe^x^-glycans affects the gene expression. Gene expression varies in different growth stages of the bacteria^36^, and thus a difference in growth rate between replicates and experiments may give varying results. When studying the response to mucins, and in particularly gene expression, the viability measurement by alamarBlue is useful when comparing different strains of bacteria to ensure that a difference between them is a cause of genetic strain differences and not a cause of occasional differences in growth. Here we showed that the correlation between binding to human gastric mucins with varying glycosylation and *babA* expression tended to be negative, and in the presence of Le^b^, the expression of the *babA* adhesin is reduced. In addition, our previous study showed a change in *babA* expression in response to mucins, with a lack of upregulation of *babA* only with mucins to which *H. pylori* bound via BabA^21^. Neither here nor in the previous study was there any significant change in *babA* expression level in response to the tumor mucin sample (referred to as P1 TS in ref. 21), to which there is only a low BabA-mediated binding, indicating that the degree of binding may be of importance for the regulation. Although a subset of human mucins may carry other glycan elements that stimulate BabA expression^21^, our results combined imply that BabA mediated binding represses *H. pylori babA* expression, inhibiting an increase in BabA in response to mucins where otherwise there may have been such increase, and decreasing it in the absence of such elements. The repression of *babA* expression in response to Le^b^ binding may thus act as a negative feedback loop. An excessive binding to mucins would allow the bacteria to be washed away along with shedding mucus, decreasing the amount of adhesin expressed would be one way to enable long term colonization.

Although presence of *babA* in the infecting strain has been associated with a more severe clinical outcome, presence of its ligand Le^b^ in the host has been associated with lower density of *H. pylori* in the stomach of infected individuals and lower level of gastritis, both in human children and in the rhesus monkey infection model^16^,^37^. These seemingly contradictory outcomes may be explained by a model where on the one hand binding to Le^b^ on mucins decrease *H. pylori* growth and bound bacteria also are removed and disseminated with the shedding of the mucus, whereas on the other hand, BabA can provide intimate adherence to glycolipids on the epithelial cell surface. Indeed, it has been reported that low producers of BabA are associated with more severe clinical outcomes compared to high producers or BabA-negative strains^38^, indicating a fine tuned balance between these aspects of virulence and host defense.

In conclusion, mucin glycosylation can directly affect *H. pylori* binding repertoire and *H. pylori* adhesin expression. Host ligand presentation on mucins appear to undergo a constant host pathogen adaptation and response process; when *H. pylori* encounters the mucins that build up the mucus layer, the pathogen binding repertoire changes in response to the mucin glycans. The host responds to infection by changing its mucins and mucin glycans in a time dependent manner^15^,^16^,^39^,^40^, which in turn provoke further adaptations of the pathogen. Furthermore, mucins have the potential to influence *H. pylori* pathogenicity by affecting its growth and expression of virulence factors. The effect of mucins is mediated by its glycan composition, which may inhibit *H. pylori* proliferation by adhesion and aggregation of bacteria, as well as by antibiotic effects. Since glycosylation of mucins differs between individuals and varies with disease status, these effects may influence the outcome of the infection.

## Methods

### Ethics statement

One tumor sample was from our well characterized mucin library, and that sample was collected in 1983 at the IMIM-Hospital del Mar, Barcelona, Spain, before the hospital had an ethics committee. The other samples were obtained after written informed consent and approval of the local ethics committee (Lund University Hospital, Lund, Sweden). The methods applied to these samples were performed in accordance with the committee’s regulations.

### Isolation of mucins

The main part of the study was performed on mucins isolated from two gastric specimens; one specimen was from a gastric adenocarcinoma tumor (intestinal type, hereafter referred to as tumor sample) and the other one from macroscopically normal antral mucosa of a tumor-affected stomach (hereafter referred to as normal sample), as evaluated by a clinical pathologist. In addition, a series of additional differentially glycosylated mucins were used for the results shown in Figs 1 and 6. Mucins were isolated using isopycnic gradient centrifugation and characterized for mucin and glycan content as previously described^21^,^23^,^41^. In this study we only used mucins present in the supernatant, where most of the MUC5AC and MUC6 molecules usually are found. The mucin content and carbohydrate structures present in the samples are summarized in Table 1.

Gradient fractions containing mucins were pooled together to obtain one sample for each specimen. All samples were extensively dialyzed in phosphate buffered saline (PBS) to remove guanidinium hydrochloride and cesium chloride (CsCl). Mucin concentration in pooled samples was determined by detection of carbohydrate as periodate-oxidisable structures in a microtiter-based assay: Flexible 96-well plates (BD Biosciences, Franklin Lakes, NJ, USA) were coated with mucin sample and left overnight at 4 °C. After washing three times with washing solution (5 mM Tris-HCl, 0.15 M NaCl, 0.005% Tween 20, 0.02% NaN_3_, pH 7.75), the carbohydrates were oxidized by treatment with 25 mM sodium metaperiodate in 0.1 M sodium acetate buffer, pH 5.5 for 20 min in room temperature. The plates were washed again and the wells were blocked with DELFIA blocking solution (50 mM Tris-HCl, 0.15 M NaCl, 90 μM CaCl_2_, 4 μM EDTA, 0.02% NaN_3_, 6% sorbitol, 0.1% BSA, pH 7.75) for 1 h. After further washing steps, the samples were incubated for 1 h with 2.5 μM biotin hydrazide in 0.1 M sodium acetate buffer, pH 5.5, followed by washing again. Europium-labeled streptavidin was diluted 1:400 in assay buffer (50 mM Tris-HCl, 0.15 M NaCl, 20 μM DTPA, 0.01% Tween 20, 0.02% NaN_3_, 1.5% BSA, pH 7.75) and was added to the wells. After 1 h incubation, the plates were washed six times and then incubated with enhancement solution (0.05 M NaOH, 0.1 M ftalat, 0.1% Triton X-100, 50 μM TOPO, 15 μM β-NTA) for 5 min on a shaker. The plates were measured using Wallac 1420 VICTOR^2^ plate reader with the Europium label protocol (PerkinElmer, Waltham, MA, USA). The concentrations were calculated from a standard curve of a fusion protein of MUC1, 16TR and IgG2a Fc starting at a concentration of 20 μg/mL and using seven 1:2 serial dilutions. This method of concentration determination was chosen as all mucins do not come into solution after freeze drying, and determining concentration by freeze drying therefore can contain large errors as well as remove mucin species selectively. Since this study focuses on the effects of carbohydrates, setting the concentration based on the carbohydrate content appear appropriate.

### Bacterial strains and culture conditions

*H. pylori* strains J99 and P12 were cultured on Brucella agar (Brucella Medium Base, Oxoid, Basingstoke, Hampshire, England) supplemented with 10% citrated bovine blood (Svenska Labfab, Ljusne, Sweden), 1% IsoVitox (Oxoid), 4 mg/L amphotericin B, 10 mg/L vancomycin and 5 mg/L trimethoprim in 5% O_2_ and 15% CO_2_ at 37 °C. Plates or broth were, when required, supplemented with streptomycin (100 mg/L), chloramphenicol (20 mg/L) or kanamycin (25 mg/L). Strain J99Δ*arsS* was created by transformation of a Δ*arsS*::*rpsLCAT* PCR fragment (P38/P43) generated by amplifying regions flanking the deletion by primers P38 with P40 and P41 with P43, the *rpsLCAT* cassette with P54 and P55 and adjoining these pieces with primers P38 and P43. Insertion and deletion of the 5′ region of the *arsS* gene was verified by PCR. The Δ*arsS*::*rpsLcat* PCR fragment was cloned in pUC19 and used as PCR template for making the other Δ*arsS* mutant strains described. *H. pylori* strains J99 wild type (wt), J99Δ*babA*, J99Δ*sabA* and J99Δ*babA*Δ*sabA* were kindly provided by Prof. Thomas Borén, Umeå University, Sweden. The P12Δ*sabAB* and Δ*babA* mutants were constructed by transformation of chromosomal DNA of J99 Δ*sabA*::Cm or J99 Δ*babA*::Cm^42^. The mutants were verified by PCR (*sabA, babA*), Immunoblot analysis (α-SabA and α-BabA) and RIA assay (SLe^x^ and Le^b^). Primers used for PCR were the following; SabA-1 and SabA-R for *sabA* locus, A81 and A19 for *babA* locus in strain P12. All primer sequences are listed in Table 2.

### RadioImmunoAssay (RIA)

Bacterial samples from at least five independent experiments, were collected from plate, washed twice in Sia buffer (1% periodate-treated BSA in PBS containing 0.05% Tween-20). Binding to soluble ^125^I-SLe^x^- and Le^b^-receptor conjugates was performed as previously described^43^. Samples were assayed in duplicates using cocktail conjugate mixtures. The percentage of conjugate bound to bacteria was calculated for every sample.

### SDS PAGE and immunoblot analysis

Bacterial samples from at least five independent experiments, were collected from plate, washed twice in Sia buffer. SDS Page analysis was made using NuPage Novex Bis-Tris protein gels (4–12% or 12%) using 1x MOPS SDS as running buffer (Life Technologies). The gels were either stained with PageBlue Protein stain (Thermo Scientific) or transferred to PVDF membrane using Trans-Blot SD Semi-Dry Transfer Cell (Bio Rad). Immunoblot analysis was performed as previously described^44^. Antibodies against AlpB (AK262)^45^, SabA (AK278) and BabA (AK277), were used in combination with secondary anti-rabbit IgG-HRP (P0160, DAKO A/S, Denmark). Blots were developed with SuperSignal West Pico Chemiluminescent Substrate (Pierce, Rockford, IL) and detected on High Performance Chemiluminescence film (GE Healthcare). BabA, SabA and AlpB protein densities were measured by ImageJ software (a public domain image processing program from the National Institutes of Health, Maryland, USA).The adhesin/AlpB density ratio for each sample was used to calculate the fold difference between the wt and the arsS deletion mutant.

### Culture of *H. pylori* with mucins and glycoconjugates

*H. pylori* were harvested from agar plates (without chloramphenicol or kanamycin) into PBS and centrifuged at 2500 × *g* for 3 min. Bacteria were then resuspended and cultured in 6 replicates in 60% Brucella broth, 20% fetal bovine serum (FBS), 20% PBS containing mucins, with a final *H. pylori* starting concentration of OD_600_ 0.2 and 50 μg/mL mucin. A control for normal proliferation was obtained by adding PBS without mucins to the culture medium. A second control was obtained by dialyzing mucin isolation buffer against PBS, parallel to the dialysis of the mucin, and this control gave very similar results to PBS alone. *H. pylori* were also cultured with 50 μg/mL of glycoconjugates of the carbohydrate structures Le^b^ and sialyl-Le^x^ coupled to human serum albumin (HSA) to create multivalency (IsoSep AB, Tullinge, Sweden). The control for normal proliferation in this assay was obtained by adding HSA (50 μg/mL) in PBS to the culture medium, or PBS alone, which gave very similar results. Bacteria were cultured in a total volume of 100 μL in 96-well plates for 24 or 48 hours at 37 °C under aerobic (20% O_2_, 5% CO_2_) or microaerobic (5% O_2_, 10% CO_2_) conditions with similar results. The strains used in this study grow well both under microaerobic and aerobic conditions. The comparisons between growth with different mucins or glycoconjugates were always performed on cultures run simultaneously, using the same starting inoculum, as slight variations in growth rate or length of lag phase may occur between experiments.

### Proliferation assay

OD at 560 nm was measured at time points throughout the culturing of *H. pylori* with mucins and glycoconjugates. In addition, alamarBlue (Molecular Probes, Leiden, The Netherlands) was added to the wells to measure the metabolic activity of the bacteria. The plates were incubated for 1 hour at 37 °C with 10 μL of alamarBlue per well and measured at OD_560_ or at both OD_540_ and OD_600_ to calculate the relative amount of reduced alamarBlue according to the manufacturer’s instructions. At the end of the incubation, bacteria from a subset of wells were cultured on Brucella plates and CFU were counted after 5 days of incubation.

### Fluorescence microscopy

Bacteria from two replicate wells were pooled after 24 and 37 h proliferation with mucin samples or glycoconjugates and stained with a LIVE/DEAD *Bac*Light bacterial viability kit (Molecular Probes) according to the manufacturer’s instructions. Stained bacteria were applied to a microscopy slide and studied immediately under a fluorescence microscope for red and green fluorescence simultaneously.

### RNA extraction, cDNA synthesis and Real-time PCR

After 24 h culture with mucins or glycoconjugates, 100 μL of RNAprotect Bacteria Reagent (Qiagen GmbH, Hilden, Germany) was added to each well and 6 wells per sample were pooled. RNA extraction was continued with Qiagen’s RNeasy kit, including DNase treatment with Qiagen’s RNase-Free DNase Set. cDNA was synthesized from 400 ng RNA using Quantitect Reverse Transcription kit (Qiagen) and real-time PCR was run as previously described^21^ with primer sequences listed in Table 3. Gene expression was calculated with 2^−ΔCt^ and normalized against the 16S rRNA expression.

### Binding assay

*H. pylori* were grown on agar plates for 24–48 h and harvested in PBS. The bacteria were centrifuged at 2500 × *g* for 3 min and then resuspended in Blocking Reagent for ELISA (Roche). Mucin samples were diluted in 4 M GuHCl and glycoconjugates of the carbohydrate structures Le^b^ and sialyl-Le^x^ coupled to human serum albumin were diluted in PBS to 4 μg/mL and coated in 4 replicates on 96-well polysorp plates (NUNC A/S, Roskilde, Denmark) overnight at 4 °C. The plates were washed three times with PBS 0.05% Tween and the wells were blocked for 1 hour with Blocking Reagent for ELISA containing 0.05% Tween (blocking buffer). After discarding the blocking buffer, bacteria with an OD_600_ of 0.1 were diluted 1:10 in blocking buffer and added to the plates, which then were incubated in a bacterial shaker at 37 °C for 2 hours. Then the plates were washed three times, which was repeated between every subsequent incubation step. The plates were incubated for 1 hour at room temperature with rabbit anti-*H. pylori* serum diluted 1:1000 in blocking buffer, and then another hour with HRP-conjugated donkey anti-rabbit IgG diluted 1:10,000 in blocking buffer. For detection, 100 μL of TMB substrate was added and the plates were incubated for 15 min before the reaction was stopped with an equivalent amount of 0.5 M H_2_SO_4_. The absorbance was measured in a microplate reader at 450 nm after color stabilization. Control wells without mucin coating but with all bacterial strains as well as wells with all mucin coatings but omitting the bacteria from the protocol above were included in all experiments. The signal in both types of control wells ranged from 0.06–0.1. Data in the figures are shown after subtracting this background signal.

### Statistical analyses

Statistical analyses were performed using Graph Pad Prism 5.0 (GraphPad Software Inc.) software package. One-Way ANOVA followed by Bonferroni’s or Dunnett’s post hoc test and student’s t-test, or Kruskal-Walllis One-way ANOVA on ranks with Dunn’s multiple comparisons test and Mann-Whitney U-test (when appropriate) were used to compare between groups. The Pearson product-moment coefficient was calculated to analyze correlation. Grubb’s test was performed to verify an outlier. The level of statistical significance was set at p ≤ 0.05.

## Additional Information

**How to cite this article**: Skoog, E. C. *et al*. BabA dependent binding of *Helicobacter pylori* to human gastric mucins cause aggregation that inhibits proliferation and is regulated via ArsS. *Sci. Rep.* **7**, 40656; doi: 10.1038/srep40656 (2017).

**Publisher's note:** Springer Nature remains neutral with regard to jurisdictional claims in published maps and institutional affiliations.

## Figures and Tables

**Table 1:** Content of mucin and carbohydrates of the mucin samples isolated from tumor and normal gastric tissue.

Origin	Sample	MUC5AC	MUC6	MUC2	MUC5B	Le^b^	Sialyl-Le^x^	Sialyl-Le^a^	α1,4-GlcNAc
Gastric tumor	Tumor	+	++	−	++	++	++	+	−
Normal mucosa of tumor-affected stomach	Normal 1	+	+	−	−	++	−	−	++
	Normal 2	++	−	−	−	++	−	−	+
	Normal 3	+	−	−	−	++	−	−	+
	Normal 4	+	−	−	−	++	−	−	−
Normal mucosa from obesity surgery	Normal 5	++	+			++			−

(normal). The relative amount of each mucin and of glycan structures relevant for interactions with *H. pylori*, as determined by ELISA, is indicated with ++ (above 75% of the highest assay value), + (5–74% of the highest assay value) or − (below 5% of the highest assay value).

**Table 2:** Primers for deletion mutants.

Primer	Sequence (5′>3′)
P38	ATGATAGAAGTTTTAATGATAGAAGATGA
P40	ATCCATAGTTATAAAGCATCTAAAAAAGATAGAGAAACGCAA
P41	ATCCATAGTTATAAAGCATCTAAAAAAGATAGAGAAACGCAA
P43	ACCTGTTTGTCATCGCTGTATTTGA
P54	TTGCGTTTCTCTATCTTTTTTAGATGCTTTATAACTATGGATTAAACAC
P55	TTATCCCCTTGAGCGAATTATCAGTGCGACAAACTGGG
SabA-1	ATGAATTCCTCTAGCAATGTGTGG
SabA-R	AACACCGCGTATTGCGTTGG
A79	CAAGAAATAAACCGCTCAT
A81	GCACCCCAGCCATTTTTCCTTA
A19	GAAGAGGTGCTTTCTTGACCATTAGCGTTACCCCGCATGCGT

**Table 3:** Primers used in real-time PCR.

Gene	Full name	Direction	Sequence	Reference
*jhpr6*	16S ribosomal RNA	Forward	TCGGATTGTAGGCTGCAACTC	21
		Reverse	CCGCAACATGGCTGATTTG	
*babA*	Blood group antigen binding adhesin A	Forward	GGAAGCGAAAGTTTGAGTGG	21
		Reverse	GAGAGGCTTAGCGGGACTTT	
*sabA*	Sialic acid binding adhesin A	Forward	GAGCGTTGCTTACGGTTGAG	21
		Reverse	CCCAACAAAACGCTACCACT	
